# Trends and advances in tumor immunology and lung cancer immunotherapy

**DOI:** 10.1186/s13046-016-0439-3

**Published:** 2016-09-29

**Authors:** Mohanad Aldarouish, Cailian Wang

**Affiliations:** Department of Oncology, Zhongda Hospital, School of Medicine, Southeast University, 87 Dingjiaqiao Rd, Nanjing, Jiangsu Province People’s Republic of China

**Keywords:** Lung cancer, Tumor immunology, Immunotherapy, Cancer vaccines, Clinical trials, Immune checkpoint inhibitors

## Abstract

Among several types of tumor, lung cancer is considered one of the most fatal and still the main cause of cancer-related deaths. Although chemotherapeutic agents can improve survival and quality of life compared with symptomatic treatment, cancers usually still progress after chemotherapy and are often aggravated by serious side effects. In the last few years there has been a growing interest in immunotherapy for lung cancer based on promising preliminary results in achieving meaningful and durable treatments responses with minimal manageable toxicity. This article is divided into two parts, the first part discusses the role of human immune system in controlling and eradicating cancer and the mechanisms of immune response evasion by tumor. The second part reviews the recent progress made in immunotherapy for lung cancer with results from trials evaluating therapeutic vaccines in addition to immune checkpoint blockade, specifically cytotoxic T lymphocyte associated protein 4, programmed death receptor 1 pathway, using monoclonal antibodies.

## Background

Lung cancer is the leading cause of cancer death and the second most common cancer in the world. According to 2012 GLOBOCAN estimation, the total number of lung cancer new cases was about 1.8 million worldwide. In China, 652842 new cases were recorded in 2012 compared with 733280 new cases in 2015 [1]. For years the standard treatment strategies of lung cancer have been surgery, chemotherapy, radiation therapy and targeted therapy [2]. Recently, tumor immunotherapy is attracting the most attention among different therapeutic options for treatment of lung cancer.

Cancer immunotherapy is a type of cancer treatment designed to boost the body’s natural defenses against cancer. It is divided into two categories, passive and active immunotherapy. Passive immunotherapy is defined as an administration of agents such as monoclonal antibodies or adaptive cell therapy that directly target tumor [3, 4]. Whereas, active immunotherapy aims to stimulate the hosts own immune system to eradicate cancer depending on vaccination with tumor antigens, non-specific immunomodulation using bacterial products, or targeting negative regulatory receptors that prevent the development of the tumor immune response [5]. Finally, it should be noted that efficient tumor immunotherapy must induce a potent anti-tumor immune response and overcome the effect of tumor immunosuppression [6].

## Cancer immunology

**Fig. 1:**
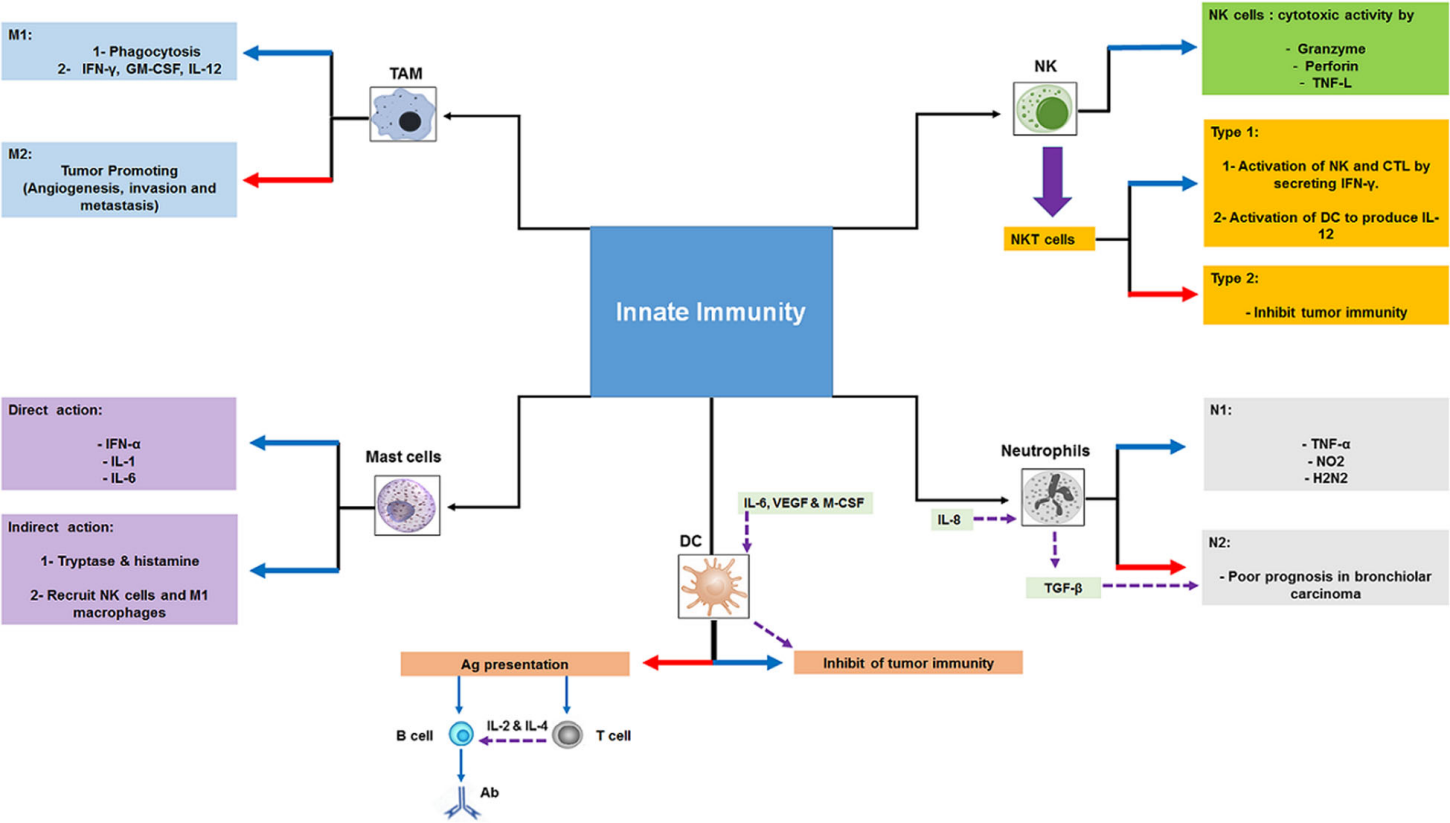
A schematic representation of the role of innate immune cell subsets in tumor immunity. Blue arrows represent the anti-tumor action, Red arrows represent the inhibition of anti-tumor immunity. TAM: Tumor Associated Macrophages, M1: Classically Activated Macrophages, M2: Alternatively Activated Macrophages, NK: Natural Killer cells, CTL: Cytotoxic Lymphocytes, VEGF: Vascular Endothelial Growth Factor, GM-CSF: Granulocyte-Macrophage Colony-Stimulating Factor, M-CSF: Macrophage Colony-Stimulating Factor, TGF-β: Transforming Growth Factor-Beta, Ab: Antibody. Note: T and B cells are related to the adaptive immune response

**Fig. 2:**
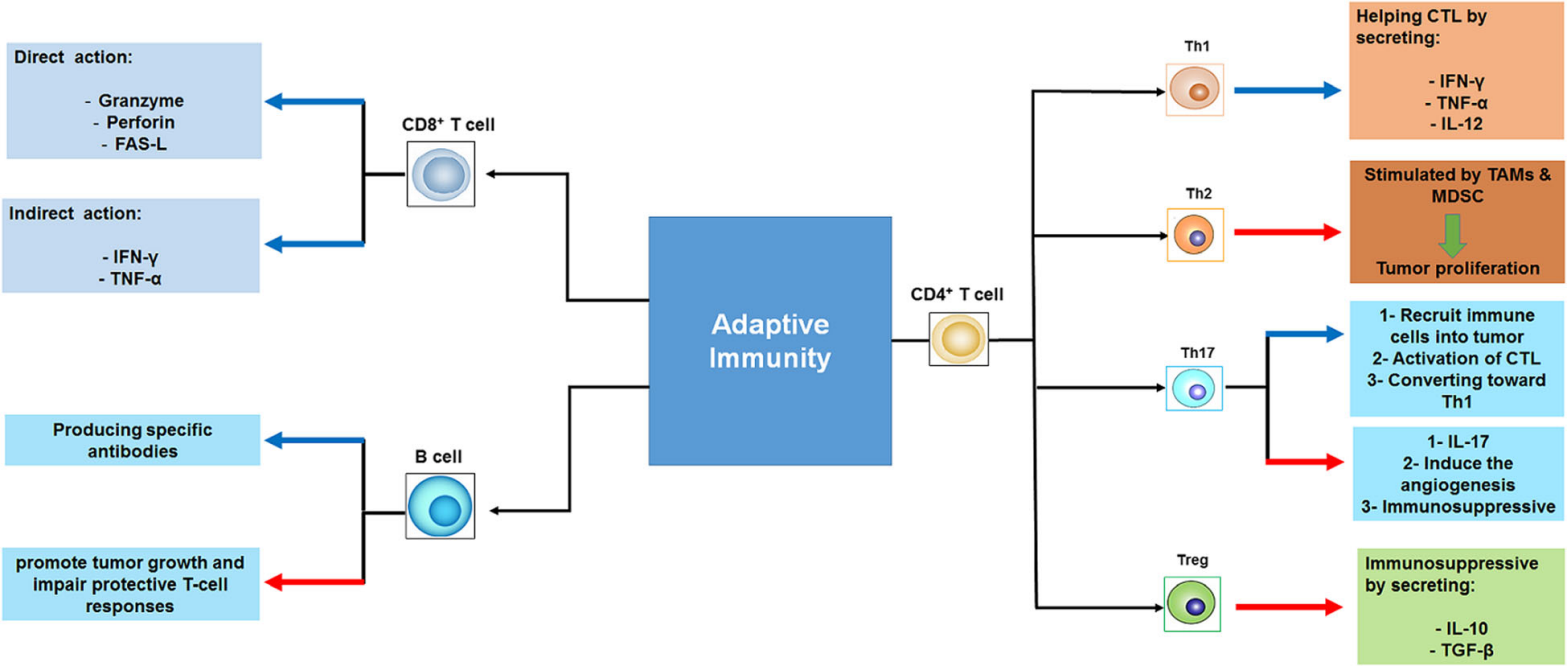
A schematic representation of the role of adaptive immune cell subsets in tumor immunity. Blue arrows represent the anti-tumor action, Red arrows represent the inhibition of anti-tumor immunity. TNF-α: Tumor necrosis factor alpha, IFN-ɣ: Interferon gamma, CTLs: CD8^+^ cytotoxic lymphocytes, TGF-β: Transforming growth factor beta, MDSC: Myeloid-derived suppressor cells

Both innate immunity (Fig. 1) and adaptive immunity (Fig. 2) play a crucial role in antitumor immune response. Innate immunity is composed of macrophages, granulocytes, mast cells, DCs and natural killer (NK) cells. Whereas, adaptive immunity is composed of B cells, CD8^+^ cytotoxic lymphocytes (CTLs) and CD4^+^ helper T cells. It must be mentioned that NK cells and ɣδ T cells play at the interface between innate and adaptive immunity [7, 8]. The roles of innate and adaptive immunity in controlling and eradicating cancers are discussed below.

CTLs are considered the backbone of immune response against tumor. Several studies have reported that tumor-infiltrating lymphocytes TILs (mononuclear cells derived from the inflammatory infiltrate in human solid tumors) contain an abundant level of CTLs with an ability to invade tumor cells which they were derived. Recognition of tumor antigen by specific T cells is a necessary prerequisite for the induction of effective antitumor immune response [9–11]. Tumor antigen presentation might be mediated by tumor cells in tumor draining lymph nodes (direct presentation) or via cross-presentation by pAPC [12]. Cross priming of naïve CD8^+^ T cells by pAPC invokes a program leading to tumor specific CTLs which proliferate and traffic to the tumor site where they ultimately attack and destroy tumor cells [13]. CTLs use various mechanisms to kill tumor cells through granzymes, perforin [14–16], and ligands of the tumor necrosis factor (TNF) superfamily such as Fas ligand [17]. The anti-tumor effect is also achieved by secretion of Interferon gamma (IFN-ɣ) [18] and TNF alpha (TNF-α) [19] from activated CD8^+^ T cells.

Understanding the importance role of CD4^+^ T cells in the antitumor immune response has grown dramatically over the past decade. Upon encountering antigens and adequate co-stimulation signals, naive CD4^+^ T cells are activated, polarized, and differentiated into distinct subsets including Th1, Th2 [20], Tregs [21], Th17, [22], Th9 [23], Th22 [24] and follicular helper T cells (T_FH_) [25]. Among these various CD4^+^ T cells subpopulations, Th1 subset plays a clear antitumor role by coordinating cell-mediated immunity against cancer cells [26]. First and foremost, Th1 cells, by producing large amounts of IFN-ɣ and chemokines, enhance the expansion, priming and infiltration of CD8^+^ T cells into the tumor site [27]. Importantly, The IFN-ɣ which secreted by Th1 cells exerts anti-proliferative, pro-apoptotic actions and inhibit angiogenesis in tumor cells in a CD8^+^ T cells-independent manner [28]. Th1 cells also recruit and activate inflammatory cells (macrophages, granulocytes, eosinophils and NK cells) in around the tumor [29]. Indeed, Th1 cells can kill MHC-II^+^ tumor cells directly through perforine and granzyme, TNF-related apoptosis inducing ligand (TRAIL) receptor and Fas/Fas ligand pathways [30].

NK cells have been gaining importance in recent years as an efficient approach in cancer immunotherapy. These cells are able to clear tumor cells directly through several mechanisms. NK cells induce the tumor cell apoptosis by 1) secretion of cytoplasmic granules, perforin and granzymes [31], 2) expression of death receptor-mediated apoptosis [32] or 3) secretion of TNF-α [33] and destroying tumor cells through antibody dependent cellular cytotoxicity by expressing CD16 [34]. Furthermore, NK cells have an indirect antitumor activity through producing cytokines, chemokines and growth factors [35]. The IFN-ɣ which produced by NK cells is responsible for induction of CD8^+^ T cells to become CTLs as well as differentiation of CD4^+^ T cells toward a Th1 response [36]. NK cells also induce inflammatory responses; modulate monocyte, DC, granulocyte growth and differentiation; and enhance subsequent adaptive immune responses through their released cytokines [37].

Macrophages play a vital role in antitumor innate response by elimination of apoptotic tumor cells in order to obviate autoimmunity. During apoptosis, tumor cells express special molecules at their surface (lipid phosphatidylserine, oxidized PS, oxidized low-density lipoprotein and the multi-functional protein calreticulin) which are recognized by macrophages and lead to tumor cell phagocytosis [38, 39]. In contrast, it has been documented that tumor-associated macrophages type 2 (M2) are responsible for tumor metastasis [40] and progression [41] by promoting the transfer of tumor cells into the local blood vessels [40] and inhibiting of tumor-specific T cells [41].

Tumor antigens have been classified into two categories, 1) tumor-associated antigens (TAAs) which are expressed by more than one type of tumor cells as well as normal tissues and, 2) tumor specific antigens (TSAs), on the other hand, are products of random somatic point mutations induced by physical or chemical carcinogens and therefore expressed uniquely by individual tumors and not by any normal tissue, representing the only true tumor-specific antigens [42, 43].

## Immune response evasion by tumor

The mechanisms that allow cancer to evade the anti-tumor immune response are mainly divided into two categories, intrinsic and extrinsic. Tumor intrinsic mechanism is achieved by immunosuppressive cytokines, vascular endothelial growth factor (VEGF), indoleamine 2,3-dioxygenase (IDO), programmed cell death ligand (PD-L), Fas ligand (Fas-L) and Treg. Tumor extrinsic mechanism is mediated by suppressive cells including alternatively activated M2-like tumour-associated macrophages (TAMs), suppressive T cells, immature APCs, plasmacytoid dendritic cells (pDCs) and heterogeneous population of myeloid-derived suppressor cells (MDSCs) [44].

Lung cancer evades the immune response through multiple mechanisms. For instance, lung cancer cells go through a slow process of immunoediting, in which precancerous cell slowly undergoes selective adaptation to oppose immune surveillance, a phenomenon called “immune sculpting” [45]. Lung cancer cells also disturb the routine processing of their antigens by pAPC through secretion of various proteins including STAT-3, IDO, TGF-β, and IL-10 [46, 47]. Besides, the dense fibrotic stroma around tumor region influences antitumor immunity by limiting access of T cells to tumor cells [48]. Moreover, lung cancer evade the host immune response by down-regulation of MHC class I molecule expression, thereby rendering any endogenous or therapeutic anti-tumor T cell responses ineffective [49]. Finally, lung cancer promotes the increase of immune suppressive cells, specifically Treg [50] and MDSC [51]. These immune suppressive cells accumulate in the tumor microenvironment, promote tumor growth, and downregulate antitumor immune responses [50, 51].

Recently it was revealed that the immune checkpoint molecules, specifically cytotoxic T-lymphocyte antigen-4 (CTLA-4) and programmed death-1 (PD-1), play an important role in tumor immune response evasion. CTLA-4 is a protein receptor expressed on the surface of CTL following their full activation. The binding between CTLA-4 and B7-1 (CD80) or B7-2 (CD86) on APCs prevents the overactivity of T cells under normal conditions. During cancer, T cells express a high level of CTLA-4, so that, cancer can evade the cytotoxic effect of T cells [52, 53]. PD-1 is a surface receptor expressed on the surface of activated T cells, B cells, NK cells, and host tissues. Binding of PD-1 with its ligand (PD-L1) on the surface of APCs leads to the tolerance of T cells [54, 55]. It has been documented that numerous epithelial cancers express PD-L1 which leads to T-cell anergy through binding with their PD-1 molecules [56].

## Lung cancer vaccines

Over the last 15 years, numerous efforts have been made to enhance a potent antitumor responses using vaccines to target specific tumor-associated antigens. Although, most studies did not reach their final goals, different subsets analysis showed that, those vaccines could an effective strategy to treat several kinds of tumor including lung cancer. Furthermore, numerous studies indicated that combination of therapeutic vaccines with immune checkpoint inhibitors may play a crucial role in the therapy of lung cancer. Finally, it’s important to mention that one of the most important aspect in immunotherapy is designing vaccine that stimulate both a potent immune response and a correlative clinical response. In the following section, we will discuss the efficacy of therapeutic vaccines that have been studied extensively in lung cancer.

### Melanoma-associated antigen A3 (MAGE-A3) based vaccine

Melanoma-associated antigen-A3 (MAGE-A3) is normally presented on testes and placenta. It is also considered as a tumor specific antigen that only expressed on the surface of tumor cells such as melanoma, bladder, non small cell lung carcinoma (NSCLC) and hepatocellular cancer [57]. It has been documented that tumor cells expressing MAGE-A3 antigen are not able to present it to the helper (CD4^+^) and CTLs. So that, this antigen could be used as immunotherapeutic agent to activate an effector immune response against tumor MAGE-A3 expressing cells [58].

In a double-blind, randomized, placebo-controlled phase II study, 182 patients of stage IB/II NSCLC were enrolled to receive the recombinant MAGE-A3 vaccine (*n* = 122) or placebo (*n* = 60). The results showed that there was no significant difference between vaccinated patients and placebo group regarding to disease-free interval (DFI), disease-free survival (DFS), and overall survival (OS) (Table 1). On the other hand, it was recorded that after 44 months, recurrence was observed in 35 % of patients who received recombinant MAGE-A3 vaccine compared with 43 % of patients in placebo group. Interestingly, IgG antibodies against MAGE-A3 has been detected in all MAGE-A3 vaccinated patients which refers to the ability of this vaccine to induce a specific immune response. These results proved the efficiency of MAGE-A3 vaccine with minimal toxicity. This study referred that MAGE-A3 vaccine could still provide benefit in combination with immune checkpoint inhibitors that reverse a tumor’s immunosuppressive effects [59].

**Table 1:** Clinical trials of therapeutic vaccines in lung cancer

Vaccine	Phase	Pt No.	Stages	Results
MAGE-A3	II	182	IB/II	DFI: (HR: 0.75, 95 % CI, 0.46 to 1.23; two-sided *P* = .254); DFS: (HR: 0.76; 95 % CI, 0.48 to 1.21; *P* = .248); OS: (HR, 0.81; 95 % CI, 0.47 to 1.40; *P* = .454) [59].
BLP25	II	171	IIIB/IV	Median OS: 17.4 months with L-BLP25/BSC vs 13.0 months with BSC alone [68]. Subset analysis (*n* = 65): Median OS: 30.6 months with L-BLP25/BSC vs 13.3 months with BSC alone [69].
	III	1239	III	Median OS: 25.6 months with L-BLP25 vs 22.3 months with placebo [70]. Subset analysis (patients with previous chemoradiotherapy): Median OS: 30.8 months with L-BLP25 vs 20.6 months with placebo [70].
Belagenpumatucel-L	II	75	II/IV	Estimated 2-years survival: 52 % with high doses vs 20 % with low doses [75].
	III	532	III/IV	Median OS: 20.3 months with belagenpumatucel-L vs 17.8 months with placebo [76]. Subset analysis (patients with pretreatment radiation): Median OS: 40.1 months with belagenpumatucel-L vs 10.3 months with placebo [76].
CIMAvax EGF	II	80	IIIB/IV	Median survival: 11.7 months with GAR vs 3.6 months with PAR [81].
TG4010	II	148	IIIB/IV	6-months PFS: 43.2 % with TG4010/chemotherapy vs 35.1 % with chemotherapy alone [84].

Abbreviations: *DFI* disease free interval, *DFS* disease-free survival, *OS* overall survival, *HR* hazard ratio, *BSC* best supportive care, *PFS* progression free survival, *GAR* good anti-EGF antibody response, *PAR* poor antibody response

**Table 2:** Results of clinical activity for immune checkpoint inhibitors in lung cancer

Target	Agent/Ab type	Pt No.	Phase	Results
CTLA-4	Ipilimumab IgG1	204	II	Phased ipilimumab: irPFS: 5.7 months; PFS: 5.1 months; Median OS: 12.2 months. Concurrent ipilimumab: irPFS: 5.5 months; PFS: 4.1 months; Median OS: 9.7 months. Control: irPFS: 4.6 months; PFS: 4.2 months; Median OS: 8.3 months [90].
	Tremelimumab IgG2	87	II	ORR: 4.8 %; PFS: 20.9 % vs. 14.3 % with supportive care [91].
		29	II	Disease control: 31 % of patients, Median PFS: 6.2 months; Median OS: 10.7 months [92].
PD-1	Nivolumab IgG4	129	I	Median OS across doses: 9.9 months; Median OS: 14.9 months at 3 mg/kg vs 9.2 at 1&10 mg/kg; ORR: 3 (1 mg/kg), 24 (3 mg/kg), and 20 % (10 mg/kg). 1-year OS rates at 3 mg/kg: 56 % [99].
		117	II	PR: 14.5 % of patients; Stable disease: 26 % of patients; Median duration: 6 months; Median OS: 8.2 months; 1-year OS rates: 40.8 % [100].
		582	III	Median OS: 12.2 months vs 9.4 months with docetaxel; ORR: 19.2 % vs 12.4 % with docetaxel. Median DOR: 17.1 months vs 5.6 months docetaxel [101].
	Pembrolizumab IgG4	495	I	ORR: 19.4 %; Median DOR: 12.5 months; Median PFS: 3.7 Months; Median OS: 12 months [102].
PD-L1	Atezolizumab IgG1	37	I	ORR: 24 %; 24-week PFS: 48 % [106].
		667	II	ORR: 19 % when atezolizumab used as a first-line therapy vs 17 % when it was a second-line or subsequent therapy [107].
		277	II	OS: 12.6 months with atezolizumab vs 9.7 with docetaxel; PFS: 2.7 months with atezolizumab vs 3 months with docetaxel; OR: 14.3 with atezolizumab vs 7.2 with docetaxel [108].
	BMS-936559 IgG4	75	I	ORR: 10 %; Stable disease ≥24 weeks: 12 %; PFS at 24 weeks: 31 % [109].
	MEDI4736 IgG1	200	I/II	ORR: 16 %; Disease control rate at 12 weeks: 42 % [110].

Abbreviations: *CTLA-4* cytotoxic T-lymphocyte antigen-4, *PD-1* programmed death 1, *PD-L1* programmed death ligand 1, *irPFS* immune-related progression-free survival, *PFS* progression-free survival, *OS*, overall survival, *ORR* objective response rate, *DOR* duration of response, *OR* objective response

To overcome the shortages of previous clinical study (small sample size and lacking of adjuvant therapy), the efficacy of MAGE-A3 vaccine was detected in phase III lung cancer which enrolled 2272 patient with NSCLC. Unfortunately, this study has been stopped in 2014 because adjuvant treatment with the MAGE-A3 immunotherapeutic did not increase disease-free survival compared with placebo in patients with MAGE-A3-positive surgically resected NSCLC [60].

### MUC1 derived liposomal BLP25 vaccine

MUC1 is a glycoprotein that expressed normally at the surface of epithelial cells in lung, stomach, intestines, eyes and several other organs and over-expressed in colon, breast, ovarian, lung and pancreatic cancers [61, 62]. It consists of four domains, extracellular subunit (20 amino acid tandem repeat domain), a small extracellular domain subunit, a transmembrane domain and a cytoplasm tail [62]. MUC1 supports tumor growth and metastasis depending on its anti-adhesive features, which prevent cell-cell adhesion [63]. The extracellular immunogenic subunit (25 A.A.) of MUC1 combined with the nonspecific adjuvant monophosphoryl lipid A and three different lipids was combined together to prepare a therapeutic lung cancer vaccine called liposomal BLP25 (L-BLP25, Stimuvax) [64, 65]. In vitro experiments showed that, stimulation of peripheral blood lymphocytes with Stimuvax resulted in induction of a strong MUC1-specific CD8^+^ T cells response [66].

In phase I; Palmer M, et al. [67], have evaluated the safety and immunogenicity of L-BLP25 vaccine in Patients with stage IIIB or IV NSCLC. They found that this vaccine could be administered with minimal toxicity and can elicit a primarily cellular immune response.

An open-label, randomized phase II trial in patients with stage IIIB or IV NSCLC who had underwent any first-line chemotherapy was undertaken to test the efficacy of L-BLP25. 171 patients from 17 centers in Canada and United Kingdom were recruited in this study. Patients were divided into two groups and received MUC1 liposomal vaccine combined the best supportive care (BSC) or only BSC, respectively. The overall survival showed a trend toward longer survival with L-BLP25 plus the BSC vs. BSC alone (median: 17.4 vs. 13.0 months) [68]. A subset analysis of patients with stage IIIb locoregional NSCLC (*n* = 65) showed a trend for improved survival in those patients treated with L-BLP25 compared with BSC alone; median OS was 30.6 versus 13.3 months [69] (Table 1).

In phase III trials; Butts C, et al. [70] started a randomized, double-blind trial called START (Stimulating Targeted Antigenic Response To NSCLC) to detect whether L-BLP25 vaccine could improve the survival in patients with stage III unresectable NSCLC when given as maintenance therapy after chemoradiation. 829 patients were received tecemotide vaccine and 410 patients were designate as placebo on a double-blind basis. The results showed that there was no statistically difference in overall survival between vaccinated patients and placebo (25.6 months vs 22.3 months). Interestingly, subgroup analysis revealed that there was a remarkable improvement in the patients who received previous concurrent chemoradiotherapy. The median overall survival for 538 (65 %) of the 829 patients assigned to tecemotide was 30.8 months compared with 20.6 months for the 268 (65 %) of 410 patients assigned to placebo (Table 1).

### Transforming growth factor-ßs (TGFßs) based vaccines

TGFß1, TGFß2, and TGFß3 are three highly homologous isoforms members of TGFß superfamily which also consists of more than 30 members (activins, NODAL, etc.) [71]. TGFß expression plays an opposite roles in cancer formation and development. In the primary stage of tumor, TGFß pathway induces cell cycle arrest and apoptosis [72, 73], whereas, in the late stage it supports tumor progression and metastasis [73, 74].

#### Belagenpumatucel-L vaccine

Belagenpumatucel-L (Lucanix^®^) vaccine aims to stimulate immune system of NSCLC patients using genetically modified and irradiated whole tumor cells that consist of a TGFß2 antisense gene [75].

In a randomized, dose-variable, phase II clinical trial; belagenpumatucel-L has been tested in 75 patients with NSCLC (stages II-IV), patients were divided into groups and received one of three doses (1.25, 2.5, or 5.0 × 10^7^ cells per injection on a monthly or every other month schedule for up to 16 injections). In the subgroup of 61 late-stage (IIIB and IV) assessable patients showed a a partial response rate of 15 %. Importantly, patients who vaccinated with high doses (≥25 × 10^6^cells per injection) showed a better OS than those who vaccinated with low doses (12.5 × 10^6^ cells per injection) with an estimated 2-years survival of 52 % versus 20 %, respectively (Table 1). Moreover, a high production level of IFN-ɣ, IL-4, and IL-6 cytokines was detected in vaccinated patients who showed a partial responses (PR) or stable diseases (SD) status. This vaccine also recorded an acceptable safety profile [75].

In phase III clinical trial, 532 patients of stage III/IV NSCLC who did not progress after platinum-based chemotherapy were divided into two groups, 270 patients were received belagenpumatucel-L and 262 patients assigned as placebo group. This trial has not achieved its endpoint to improve the overall survival (20.3 months with belagenpumatucel-L vs 17.8 months with placebo). Besides, There were no differences in PFS between two groups (4.3 months with belagenpumatucel-L vs 4.0 months with placebo). Although the overall survival was not improved, the subgroup analysis showed that patients with confirmed pretreatment radiation had a median OS of 40.1 months with belagenpumatucel-L compared with 10.3 months for placebo patients. More importantly, Patients with non-adenocarcinoma who were randomized within 12 weeks of the completion of chemotherapy had a median OS of 19.9 months on belagenpumatucel-L, while those who received placebo had an OS of 12.3 months (Table 1). These data, along with a strong safety profile, support the continued development of belagenpumatucel-L for this indication,” the investigators concluded, in spite of the study’s failure to meet its endpoint [76].

#### CIMAvax EGF vaccine

Epidermal growth factor receptor (EGFR) is a member of receptor tyrosine kinases (RTKs) family which consists of EGFR (ErbB1, HER1), ErbB2 (HER2), ErbB3 (HER3) and ErbB4 (HER4). EGFR tyrosine kinase is activated after binding with EGF and can induce a conformational receptor changing by (homo or heterodimer formation), this process is followed by substrate phosphorylation via activated EGFR and activation of downstream pathways which are responsible for cell survival and proliferation [77]. EGFR gene mutation (overactivation) leads to transformation of normal cells to be a malignant cells. The hallmarks of this transformation are, apoptosis suppression, cell proliferation, angiogenesis, metastasis, tumor-induced proinflammatory and immunosuppressive processes [78, 79]. It has been documented that overexpression of EGFR is associated with lung cancer [80].

CIMAvax EGF vaccine was developed by Cuban researchers to treat NSCLC adult patients with stage IIIB/IV after receiving conventional first-line chemotherapy through enhancing the own immune system to produce anti EGF antibodies and decrease the serum EGF. In phase II study; 80 NSCLC patients, after finishing first-line chemotherapy, were randomly assigned to receive BSC or EGF vaccine. 51.3 % of vaccinated patients showed an obvious anti-EGF antibody response (good anti-EGF antibody response -GAR-) and in none of the control group (poor antibody response -PAR-) (Table 1). Furthermore, the concentration of Serum EGF was obviously decreased in 64.3 % of vaccinated patients. Interestingly, the researchers found that there was a strong relationship between survival rate and elevation of antibody response and decreasing of serum EGF. CIMAvax EGF vaccine also recorded a good safety profile [81].

### TG4010 vaccine

TG4010 is a therapeutic lung cancer vaccine targeting MUC1 antigen. It consists of attenuated Ankara virus which modified to express MUC1 and IL-2 [82]. It aims to overcome the inhibition status of T-cell response resulted by cancer-associated MUC1 [83].

In phase II clinical trial; 148 patients with stage IIIB/IV NSCLC were divided into two groups, 74 patients received TG4010 combined with chemotherapy (cisplatin–gemcitabine), other 74 patients were received chemotherapy alone. The results revealed that, 6-month PFS was 43.2 (32/74) in the TG4010 plus chemotherapy group, and 35.1 % (26/74) in the chemotherapy alone group (Table 1). Interestingly, PFS in patients with a normal level of activated natural killer cells (aNK), was obviously higher than control group (58 % vs 38 %). Besides, the median OS in patients with normal level of aNK cells was higher than patients with high level (18 months vs 11.3 months). The authors concluded that TG4010 could improve the effect of chemotherapy in advanced NSCLC [84].

## Immune checkpoint inhibition

Several studies indicated that immune checkpoints blockade is a very promising in treating a variety of malignancies including lung cancer. Among these immune checkpoint are CTLA-4, PD-1 and PD-L1. Below is a discussion of recent progress made in immunotherapy for lung cancer using immune checkpoint inhibitors.

### Inhibition of cytotoxic T-lymphocyte-associated protein 4 (CTLA-4)

**Fig. 3:**
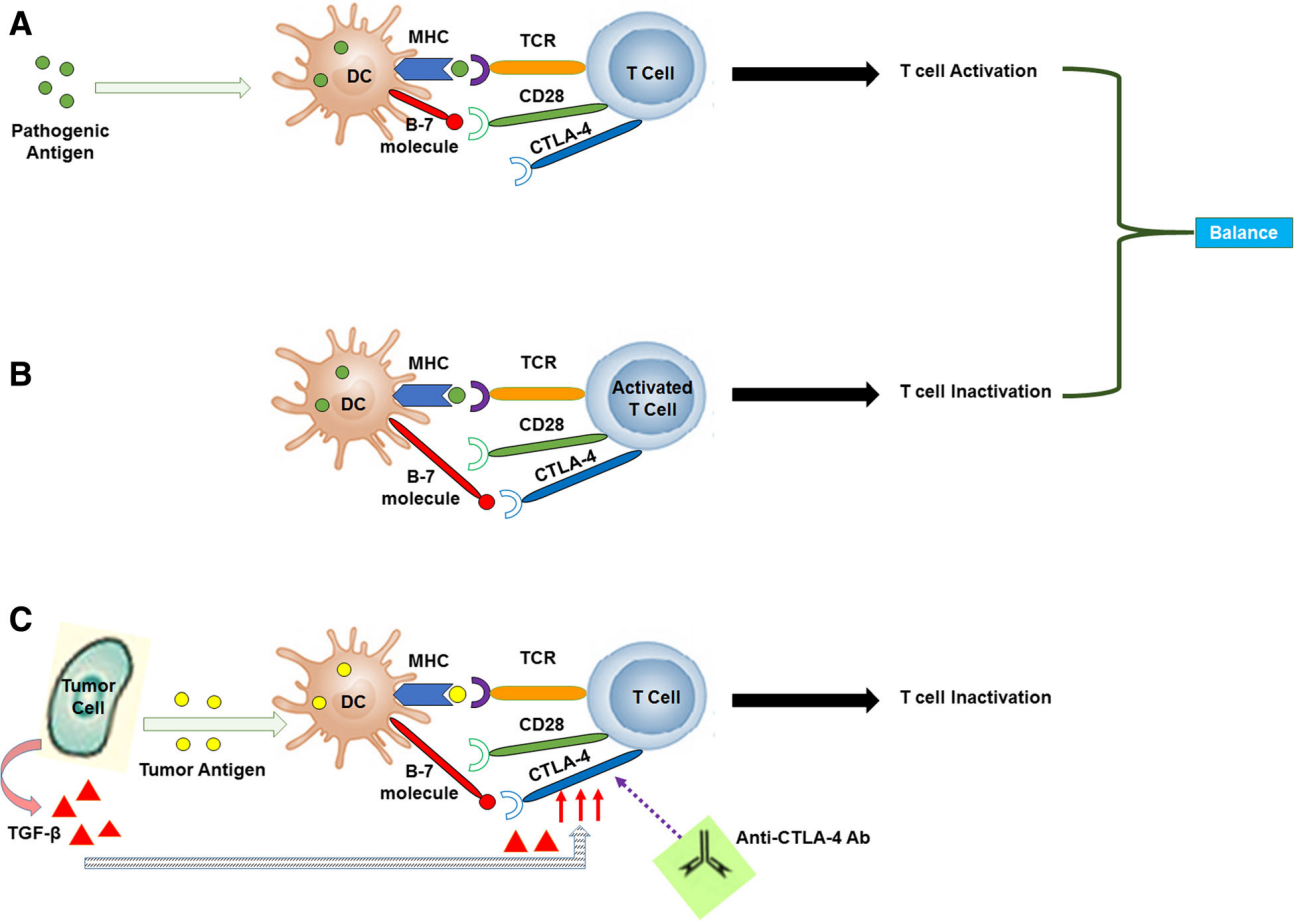
T cell activation and inactivation mechanism. **a** upon infection the full activation of specific T cell immune response requires two signals; binding of MHC/Ag complex on the APC (ex: DC) with T cell receptor (TCR) and interaction of B7-molecules (CD80/86) to their ligand (CD28) on the surface of T cell. **b** Activated T cell expresses a surface immunosuppressive molecule called CTLA-4 which compete with CD28 molecule to bind the B7 molecules. The balance between activation and inactivation signals keeps cytotoxic activity in check, while allowing T-cell function to work in a self-limited manner. **c** Tumor cells produce a suppressive cytokine that lead to the upregulation of CTLA-4 on the surface of T cells. This mechanism allows tumor cells to evade the cytotoxic effect of T cells

In order to recognize and eliminate tumor cells, CTLs require two activating signals, the first signal is provided by TAAs presented by class I molecules on pAPCs [85]. The second signal is called “costimulatory signal” which is achieved by binding of costimulatory receptor CD28 on T cells with two costimulatory molecules, B7-1 (CD80) and B7-2 (CD86) on APCs (Fig. 3a) [86]. Once a CTL becomes activated it expresses a fundamental immunosuppressive molecule called CTLA-4 on its surface which then binds with costimulatory molecules on APCs about 20 times more avidly than does CD28 (Fig. 3b). The balance between activation and inactivation signals keeps cytotoxic activity in check, while allowing T-cell function to work in a self-limited manner [87]. It has been documented that one of the most important tumor immune evasion mechanisms is upregulation of CTLA-4 expression on T cells with the help of TGF-β during the early stage of tumorigenesis. In recent years significant progress has been made in developing of specific monoclonal antibodies to inhibit CTLA-4 as a potent strategy in cancer immunotherapy (Fig. 3c) [88, 89].

#### CTLA-4 inhibition by ipilimumab

Ipilimumab (MDX-010) is a fully human IgG1 monoclonal antibody targeting CTLA-4-mediated T-cell suppression to enhance a potent immune response against tumors. In double-blind, multicenter phase II clinical trial; depending on the treatment type, NSCLC patients (*N* = 204) were divided into three arms of trial, 1) control (chemotherapy with placebo), 2) concurrent ipilimumab (ipilimumab plus chemotherapy, then, placebo plus chemotherapy) or 3) phased ipilimumab (placebo plus chemotherapy, then, ipilimumab plus chemotherapy). Patients were received treatment intravenously every 3 weeks for 18 weeks. The immune-related progression-free survival (irPFS) was assessed as the main endpoint besides other endpoints, progression-free survival (PFS), best overall response rate (BORR), immune-related BORR (irBORR), overall survival (OS), and safety [90].

It has been shown that, phased ipilimumab improved irPFS and PFS compared with control group, the median irPFS associated with phased ipilimumab was 5.7 months and the PFS was 5.1 months. Whereas, low irPFS and median PFS were seen in concurrent ipilimumab (irPFS: 5.5 months, PFS: 4.1 months), and control treatments (irPFS: 4.6 months, PFS: 4.2 months). phased ipilimumab also induced 32 % of irBORR compared with concurrent ipilimumab and control treatments (21 and 18 %, respectively). The median OS associated with phased ipilimumab was 12.2 months versus 9.7 and 8.3 months for concurrent ipilimumab and control groups, respectively (Table 2). The following adverse events (AEs) were similar across study arms, fatigue, alopecia, nausea, vomiting, and peripheral sensory neuropathy. Whereas, rash, pruritus, and diarrhea, showed a trend for increased incidence in the ipilimumab-containing arms than in chemotherapy arm [90].

These promising results lead to “A Randomized, Multicenter, Double-Blind, Multinational, phase III trial” (NCT01285609) in NSCLC which started in 2014 to determine whether the combination of ipilimumab and chemotherapy could extend the life of patients with NSCLC compared with chemotherapy alone, as well as, detecting the PFS and OS among enrolled patients. Results are expected to be revealed in late of 2018.

#### CTLA-4 inhibition by tremelimumab

Tremelimumab (ticilimumab) is a fully human IgG2 monoclonal antibody with high affinity to CTLA-4. In open-label phase II trial; tremelimumab was tested in 87 patients with NSCLC compared with supportive care only following 4 cycles of chemotherapy. PFS in tremelimumab treated patients was 20.9 % compared with 14.3 % in supportive care group (Table 2). The results revealed that 20 % of patients experienced a grade 3/4 AEs, the most common being colitis [91].

In an open-label, single-arm, phase II trial; 29 patients with advanced mesothelioma were received at least one dose of tremelimumab. This trial did not reach its primary endpoint in which only two patients had a durable partial response. On the other hand, a disease control was noted in 31 % of patients with a median PFS of 6.2 months and median OS of 10.7 months (Table 2). 27 patients experienced a grade 1/2 AEs (cutaneous rash, pruritus, colitis, or diarrhea), and 4 patients experienced at least one grade 3/4 AEs (two gastrointestinal, one neurological, two hepatic, and one pancreatic). The authors concluded that tremelimumab could be an effective treatment strategy in previously treated patients with advanced malignant mesothelioma [92].

Currently, tremelimumab is tested in a randomized phase II trial for advanced mesothelioma (NCT01843374) and in combination with other checkpoint inhibitors for treatment of NSCLC (NCT01843374) [93].

### PD-1/PDL-1 pathway

PD-1 (CD279) is a surface receptor on activated T cells, B cells, monocytes, NK cells, and many tumor infiltrating lymphocytes (TILs). Its ligand, PD-L1 (B7-H1; CD274) is expressed on the surface of resting T cells, B cells, DCs, macrophages, vascular endothelial cells, and pancreatic islet cells [94]. The binding between PD-1 and PD-L1 leads to transmitting of an inhibitory signal into T cell which reduces cytokines production and suppresses T cell proliferation [95]. This pathway plays a crucial role in protecting own body against tissue damage during response to infections [96].

**Fig. 4:**
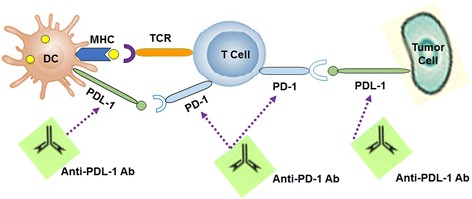
The mechanism of PD-1/PD-L1 pathway. Programmed cell death-1 (PD-1) receptor is expressed on the surface of activated T cells, B cells, monocytes, NK cells, and many TILs. Its ligand, PD-L1 is expressed on the surface of resting T cells, B cells, dendritic cells, macrophages, vascular endothelial cells, and pancreatic islet cells. The binding between PD-1 and PD-L1 leads to transmitting of an inhibitory signal into the T cell which reduces cytokine production and suppresses T-cell proliferation. PDL-1 is over expressed on tumor cells by which PD-1/PD-L1 interaction inhibits the proliferation, survival, and effector function of CTL and thus induces apoptosis of tumor-infiltrating T cells

It has been found that PDL-1 is over expressed on tumor cells or on non-transformed cells in the tumor microenvironment [95] in which PD-1/PD-L1 interaction inhibits the proliferation, survival, and effector function of CTL and thus induces apoptosis of TILs (Fig. 4) [97]. Moreover, PD-L1 molecule plays an important role in differentiation of Treg and maintaining their suppressive function. Recently, the development of anti-PD agents has taken center stage in cancer immunotherapeutic strategies [98].

#### Inhibition of PD-1 by nivolumab

Nivolumab (marketed as Opdivo) is a human monoclonal IgG4 against PD-1. In a phase I study of NSCLC; 129 patients were treated with nivolumab every 2 weeks (doses: 1, 3, or 10 mg/kg). The median OS across doses was 9.9 months in all patients. The median OS in patients receiving nivolumab 3 mg/kg was 14.9 months compared with 9.2 months in both the 1 and 10 mg/kg cohorts. Across all dose levels, 1-, 2-, and 3-year survival rates were 42, 24, and 18 %, respectively. Whereas, At the 3-mg/kg dose, 1-, 2-, and 3-year OS rates were 56, 42, and 27 %, respectively. The objective response rates (ORR) by dose were 3 (1 mg/kg), 24 (3 mg/kg), and 20 % (10 mg/kg). Among 22 patients (17 %) with objective responses, estimated median response duration was 17 months with 20.6 months of PFS. The most common treatment-related AEs were fatigue, decreased appetite, and diarrhea. Besides, treatment-related select AEs of any grade were observed in 41 % of patients and the most common included skin, GI, and pulmonary events. The authors concluded that nivolumab can induce a durable response and prolong the survival rate in patients with NSCLC [99].

In a phase II, single arm trial, was performed at 27 sites in France, Germany, Italy, and USA. 117 patients with refractory stage IIIB or stage IV squamous NSCLC were administered by intravenous nivolumab (3 mg/kg) every 2 weeks until progression or unacceptable toxic effects. Nivolumab treatment induced a partial response in 14.5 % of patients, including two patients with nontarget baseline central nervous system metastases, with a reduction in tumor burden of at least 50 % for 65 % of these responding patients. Furthermore, nivolumab treatment resulted in stable disease in 30 (26 %) patients, with a median duration of 6 months. Median overall survival was 8.2 months and overall survival at 1 year was 40.8 % (Table 2). More importantly, the results indicated that nivolumab had activity in patients with PD-L1–negative and positive tumors. Nivolumab also exhibited a manageable safety profile [100].

In phase III trial; 582 previously treated patients with advanced or metastatic squamous cell NSCLC were divided randomly into two groups, 292 patients were vaccinated subcutaneously with nivolumab every 2 weeks 3 mg/kg, other 290 patients were treated with standard chemotherapy (docetaxel/75 mg/m^2^/every 3 weeks). The OS of patients who treated with nivolumab was 12.2 months versus 9.4 months in patients who treated with chemotherapy. Moreover, nivolumab improved the ORR compared with docetaxel (19.2 % vs 12.4 %). The median duration of response induced by nivolumab was higher than that in docetaxel treated patients (17.1 months vs 5.6 months) (Table 2). Treatment-related AEs were low in severity with nivolumab and were less frequent with nivolumab than with docetaxel. The most frequently reported treatment-related AEs of any grade in the nivolumab group were fatigue, nausea, decreased appetite, and asthenia. Moreover, treatment-related serious AEs were less frequent in the nivolumab group than in the docetaxel group. The following treatment-related select AEs of any grade showed a trend for increased incidence in the nivolumab group, were rash, pruritus, erythema, diarrhea, hypothyroidis, increased alanine aminotransferase level, increased aspartate aminotransferase level, and pneumonitis. Based on these significant results, the FDA approved nivolumab (Opdivo®), made by Bristol-Myers Squibb (BMS), for treatment of advanced squamous NSCLC patients who have stopped responding to chemotherapy [101].

#### Inhibition of PD-1 by pembrolizumab

Pembrolizumab (also called: MK-3475, Keytruda) is a humanized antibody against PD-1 receptor. In phase I trial, 495 patients with advanced NSCLC were treated with pembrolizumab every 2 weeks or every 3 weeks with two different doses, 2 mg/Kg or 10 mg/Kg. Among patients, 182 were assessed as a training group and 313 as a validation group. Interestingly, antitumor activity were detected in treated patients without a serious side effects, ORR was 19.4 %, and the median duration of response was 12.5 months. The median duration of PFS was 3.7 months, and the median duration of OS was 12.0 months (Table 2). Pembrolizumab had an acceptable side-effect profile by which the most common treatment-related AEs were fatigue, pruritus, and decreased appetite. The only treatment-related AEs of an inflammatory or immune-mediated nature that occurred in more than 2 % of patients were infusion-related reactions, hypothyroidism, and pneumonitis [102].

Other primary data showed that combination of nivolumab and pembrolizumab could be an effective strategy to treat NSCLC patients. In September 2015, The FDA granted and approved pembrolizumab to treat patients with advanced, PD-L1-positive NSCLC [103, 104].

#### Inhibition of PD-L1 by Atezolizumab

Atezolizumab (also called: MPDL3280A) is a humanized IgG1 antibody that targets PD-L1 [105]. The results of phase I clinical trial in previously treated NSCLC patients showed a 24 of ORR and 48 % of 24 weeks PFS. Biomarker data analysis proved a correlation between PD-L1 status and efficacy of atezolizumab [106] (Table 2).

Phase II clinical trials were performed in two different studies, the first single-arm, phase II (BIRCH) study enrolled 667 patients with stage IIIB/IV or recurrent NSCLC to receive a treatment with atezolizumab as the first line of therapy or a subsequent therapy. 19 % of ORR was recorded when atezolizumab used as a first-line therapy compared with 17 % when it was a second-line or subsequent therapy (Table 2). The most commonly reported AEs were fatigue and nausea. Besides, the authors noted that the efficacy of Atezolizumab is associated with the high level of PD-L1 ligand [107].

In the second phase II, multicentre, open-label (POPLAR) trial, two groups of patients with NSCLC were assessed to detect the efficacy of atezolizumab, the first group consisted of 142 patients and received 1200 mg of atezolizumab once every 3 weeks, the second group assessed 135 patients and treated with 75 mg/m^2^ of docetaxel once every 3 weeks. The OS was 12.6 months in patients who received atezolizumab, compared with 9.7 months in those who received docetaxel. PFS was similar between groups (2 · 7 months with atezolizumab vs 3 months with docetaxel). Importantly, OR with atezolizumab were durable, with a median duration of 14.3 months versus 7.2 months for docetaxel (Table 2). Furthermore, it has been noted that increasing improvement of OS was associated with high expression of PD-L1. A good safety profile and toleration were recorded in atezolizumab treated patients compared with those who treated with docetaxel. 40 % of patients in the atezolizumab group experienced grade 3–4 adverse events versus 53 % in the docetaxel group. The most common atezolizumab-related grade 3 AEs were pneumonia and increased aspartate aminotransferase. No atezolizumab-related grade 4 AEs were reported. This study also confirmed the results of BIRCH trial results which showed that atezolizumab has a good efficacy in patients with the highest levels of PD-L1 [108].

In February 2015, the FDA approved atezolizumab to treat PD-L1–positive NSCLC patients who has not showed a response to chemotherapy. The randomized phase III trial for this indication is in-progress (NCT02486718).

#### Inhibition of PD-L1 by BMS-936559

BMS-936559 is a human IgG4 monoclonal antibody with high affinity to PDL1. In a multicenter phase I trial; 75 patients with NSCLC were received BMS-936559 every 2 weeks with escalating doses for a maximum of 2 years. The results showed that 10 of treated patients achieved a partial response, and 12 % had stable disease for at least 24 weeks. PFS at 24 weeks was 31 %. However, further results from this study are pending. Drug-related AEs were observed in 39 % of patients and included rash, hypothyroidism, hepatitis, and one case each of sarcoidosis, endophthalmitis, diabetes mellitus, and myasthenia gravis [109].

#### Inhibition of PD-L1 by MEDI4736

MEDI4736 (Durvalumab) is an IgG1 antagonist antibody, that blocks PD-L1 binding to PD-1 and CD80, designed with a mutated FC domain to prevent antibody-dependent cell mediated cytotoxicity (ADCC). In phase I/II, multicenter, open-label study, 228 patients (126 non-squamous and 102 squamous histology) were enrolled to evaluates the safety and clinical activity of durvalumab. The results showed that, of 200 evaluable treated patients the ORR was 16 % (27 % in PD-L1^+^), and disease control rate at 12 weeks was 42 %. The ORR in 88 patients with squamous NSCLC was 21 % whereas, in 112 with non-squamous NSCLC, the ORR was 13 % (Table 2). The most frequently Drug-related AEs were fatigue, decreased appetite and nausea [110].

Based on encouraging activity in early trials, durvalumab is currently being evaluated in several phase III trials in NSCLC such as ARCTIC, a global, phase III, randomized, open-label multicenter study (NCT02352948) which aims to asses the 1) safety and clinical activity of durvalumab versus standard of care (SoC) in patients with PD-L1^+^ tumors; and 2) the combination of durvalumab plus tremelimumab or either agent as monotherapy versus SoC in patients with PD-L1^−^ tumors [111].

## Conclusions

In the last few years there has been a growing interest in cancer immunotherapy due to its promising results in achieving significance and durable treatments responses with minimal manageable toxicity. Cancer immunotherapy has many advantages over chemotherapy or radiotherapy. In this regard, immunotherapy is receiving a particular interest due to its favorable benefits, low risk ratio and durable activity. It also showed a significant advantage by controlling tumor growth after patients stop responding to the standard treatments. One of the most important future directions in cancer immunotherapy is identifying predictive markers which can predict the antitumor effect and survival benefit before the implementation of immunotherapies. Combination therapy is another important approach in tumor therapy. Further investigations are needed to evaluate the role of combination the immunotherapeutic agents with one another and with chemotherapy, targeted therapy or other treatment options to treat cancers.

## Abbreviations

AEs: Adverse events
CTLA-4: Cytotoxic T-lymphocyte antigen-4
CTLs: CD8^+^ cytotoxic lymphocytes
DCs: Dendritic cells
DFI: Disease-free interval
DFS: Disease-free survival
EGF: Epidermal growth factor
IFN-ɣ: Interferon gamma
MAGE-A3: Melanoma-associated antigen-A3
MDSCs: Myeloid-derived suppressor cells
NK cells: Natural killer cells
NSCLC: Non small cell lung carcinoma
OS: Overall survival
pAPCs: Professional antigen presenting cells
PD-1: Programmed death-1
PD-L1: Programmed death ligand-1
TAAs: Tumor-associated antigens
TAM: Tumour-associated macrophages
Treg: Regulatory T cell
